# An Update on the Current Genomic Landscape of Breast Implant-Associated Anaplastic Large Cell Lymphoma

**DOI:** 10.3390/cancers13194921

**Published:** 2021-09-30

**Authors:** Sean Harrop, Neha Mehta-Shah, Criselle Dsouza, Ella Thompson, Anand Deva, Henry Miles Prince

**Affiliations:** 1 Peter MacCallum Cancer Centre, Melbourne, VIC 3000, Australia; Criselle.Dsouze@petermac.org (C.D.); ella.thompson@pertermac.org (E.T.); miles.prince@petermac.org (H.M.P.); 2 Division of Oncology, Washington University School of Medicine, St. Louis, MO 63110, USA; mehta-n@wustl.edu; 3 Sir Peter MacCallum Department of Oncology, University of Melbourne, Melbourne, VIC 3000, Australia; 4 Department of Plastic and Reconstructive Surgery, Macquarie University and the Integrated Specialist Healthcare Education and Research Foundation, Sydney, NSW 2006, Australia; Anand.Deva@ishc.org.au; 5 Epworth Healthcare, Melbourne, VIC 3000, Australia

**Keywords:** breast implants, epigenetic, JAK/STAT

## Abstract

**Simple Summary:**

Breast implant-associated lymphoma is a unique entity that arises in the setting of breast prostheses due to a complex interplay of external and internal factors. Understanding of the mechanisms of pathogenesis is yet to be fully elucidated but recurrent mutations in signalling pathways, tumour suppressors and epigenetic regulators have been reported. This article summarises the key studies to date that have described these genetic aberrancies, which have provided an insight into potential pathways to lymphogenesis.

**Abstract:**

Breast implant-associated lymphoma (BIA-ALCL) is a rare subtype of anaplastic large-cell lymphoma associated with breast prostheses. Most patients present with a localised periprosthetic effusion and are managed with removal of the implant and surrounding capsule. Less commonly, the lymphoma can form a mass associated with the capsule and rarely can present with disseminated disease. Recent series characterising the genomic landscape of BIA-ALCL have led to insights into the mechanisms of lymphomagenesis. Constitutive JAK/STAT pathway activation has emerged as a likely key component while, more recently, aberrancies in epigenetic regulators have been reported. This review describes the genomic characterisation reported to date and the insight these findings have provided into this rare entity.

## 1. Introduction

Anaplastic large cell lymphomas (ALCL) are a form of T-cell non-Hodgkin lymphoma that have three distinct subsets: systemic anaplastic large cell lymphoma (sALCL), primary cutaneous anaplastic lymphoma and breast implant-associated anaplastic large cell lymphoma (BIA-ALCL). ALCL can be subdivided according to the presence or absence of rearrangements of the ALK receptor tyrosine kinase (ALK) [1]. BIA-ALCL is a rare T-cell lymphoma that occurs adjacent to breast prostheses. First reported in 1997, there has now been over 1000 cases reported with recognition by the World Health Organisation as a provisional entity in 2016 [1,2,3]. Outcomes are generally favourable, with most patients presenting with an isolated periprosthetic effusion; however, a minority of patients present with disseminated disease [4]. While BIA-ALCL shares morphological and immunophenotypical features with sALCL, there is an absence of molecular aberrancies typically observed in sALCL such as rearrangements of *ALK, DUSP22* and *TP63* [5,6]. Recently, the genomic landscape of BIA-ALCL has begun to be characterised, providing insight into the oncogenic mechanisms of a rare and unique entity. Next generation sequencing (NGS) has demonstrated frequent mutations in JAK/STAT activation and signalling pathways, while recurrent mutations in key epigenetic modifiers such as *KMT2C* and *CREBBP* have now been reported (Table 1) [5,7,8,9]. This review aims to summarise the genomic landscape that has been reported to date and current understanding of the molecular aberrancies of BIA-ALCL.

## 2. Genomic Characterisation of BIA-ALCL

### 2.1. JAK/STAT Pathway Mutations

Similar to other forms of ALCL, mutations of the genes involved in the JAK/STAT pathway are frequently observed in BIA-ALCL and are suspected to play a role in lymphogenesis. Constitutive activation of the JAK/STAT pathway leads to the overexpression of key oncogenes such as *TNFRSF8* (encoding the activation marker CD30) and *IL2RA* resulting in cellular proliferation [12]. In sALCL with *ALK* rearrangement (ALK-positive sALCL), JAK/STAT pathway activation is the direct result of ALK-mediated activation [13]. In contradistinction, multiple molecular mutations have been identified as responsible for JAK/STAT pathway activation in ALK-negative sALCL, including gain-of-function mutations in *JAK1* and *STAT3* and loss-of-function mutations in negative regulators such as *SOCS1* and *SOCS3* [14]. Indeed, in BIA-ALCL, recurrent *JAK1* and *STAT3* mutations leading to activation of the JAK/STAT pathway are frequently demonstrated and STAT3 phosphorylation is uniformly demonstrated in contrast to the more heterogeneous STAT3 phosphorylation seen in ALK-negative sALCL [5,7,8,10]. JAK/STAT pathway mutations in BIA-ALCL were first identified by Blombery et al. who performed whole exome sequencing (WES) in two patients, demonstrating pathogenic somatic variants in *STAT3* and *JAK1*. The *STAT3* variant was a missense mutation with amino acid substitution S614R, affecting the SH2 domain, while the *JAK1* variant was G1097V, a known activator of STAT3. Furthermore, a germline variant in *JAK3* was reported, suggesting a potential underlying predisposition [15]. The same group then undertook a larger study of 11 patients with localised disease with no nodal or visceral involvement. There was remarkable genomic uniformity with activating mutations in the JAK/STAT pathway in ten patients (91%) with *STAT3* pathogenic variants the most frequent, detected in seven patients (64%). A loss-of-function mutation in a negative regulator of the JAK/STAT pathway, *SOCS1*, was identified and predicted to lead to pathway activation [7].

Oishi et al. identified deleterious mutations in the JAK/STAT pathway using NGS with *STAT3* and *JAK1* frequently implicated. They performed sequencing on 15 patients, with *STAT3* variants identified in 3 patients (20%). Gain of function *STAT3* Y640F was seen in two patients, with the other an activating *STAT3* S614R. One *JAK1* mutation was reported, *JAK1* G1097D, a known activator of STAT3 [5]. Di Napoli et al. also identified a gain of function *STAT3* S614R variant in a series of five patients [10]. The largest series to date is a French series by Laurent et al., which reported the results of targeted sequencing and/or WES in 22 patients, with 14 (64%) demonstrating alterations in JAK/STAT pathway members. *STAT3* (41%) and *JAK1* (18%) were the most frequent JAK/STAT pathway mutations. Missense mutations of *STAT3* at S614R were seen (as per the prior series reported), with variants at G618R, D661Y, and I659L identified. *STAT3* Y640F, an activating mutation and the most frequent variant in sALCL, has not been reported so far in any series published to date [14]. Loss of function of the negative regulatory proteins SOCS1 and SOCS3 was also reported. A recent series of nine patients by Quesada et al. again demonstrated frequent JAK/STAT pathway aberrancies, with mutations seen in *JAK1*, *JAK2*, *STAT3*, and *STAT5B*. Variants involving *JAK1* G1097 were the most common alteration identified in 44% of cases.

### 2.2. Mutations in Epigenetic Regulators

Epigenome mutations have been identified in BIA-ALCL, with *DNMT3A* and *SETD2* mutations reported in earlier series [7,10]. DNMT3A is a DNA methyltransferase that catalyses the addition of methyl groups to CpG islands in DNA and is frequently altered in PTCLs. SETD2 is the histone H3 lysine 36 methyltransferase (H3K36me3) responsible for chromatin activation and is thought to interact with p53 and regulate downstream genes [16]. Laurent et al. reported frequent mutations of epigenetic regulators (74% of patients) with loss-of-function *KMT2C*, *KMT2D*, *CHD2* and *CREBBP* mutations being common [8]. The absence of H3K4 methylation in *KMT2C* and *KMT2D* mutants was demonstrated by immunohistochemistry. *KMT2D* and *KMT2C* (previously known as *MLL2* and *MLL3*, respectively) encode histone H3K4 methyltransferases and are frequently mutated in diffuse large B-cell lymphoma and follicular lymphoma. KMT2D functions as a tumour suppressor and KMT2D deficiency impedes B-cell differentiation and B-cell signalling pathways. Mutations have also been seen in T-cell lymphomas, approximately 25% and 36% of patients with the angioimmunoblastic T-cell lymphoma and peripheral T-cell lymphoma-not otherwise specified (PTCL-NOS) subtypes [17]. *CHD2* encodes for a chromodomain helicase thought to influence the epigenome via chromatin modification, playing a role in the regulation of hematopoietic stem cell differentiation [18]. CREBBP belongs to the KAT3 family of histone/protein lysine acetyltransferases and catalyses histone acetylation. *CREBBP* mutations are highly recurrent in B-cell lymphomas and inactivate the histone acetyltransferase domain. CREBBP plays a critical role in supporting p53-dependent tumour suppressor functions [19,20]. Quesada et al. also demonstrated a high frequency of alteration in epigenetic regulatory pathways, with alteration seen in 56% of patients with *TET2, TET3, ARID4B, KDM5C, KDM6A, KMT2C/D* and *SMARCB1* mutations reported [9]. Mutations in epigenetic regulators are frequent in T-cell lymphomas and while the contribution epigenetic dysregulation makes in BIA-ALCL lymphogenesis remains unclear, the frequency in which alterations are present may have diagnostic utility.

### 2.3. Germline Mutations of Potential Relevance

Further research is needed to delineate the relative contributions of environmental factors and genetic risk, but it is notable that germline mutations in *TP53* have been detected [7,8,9,10]. Germline *TP53* mutations are associated with hereditary cancer syndromes and confer an increased risk to a variety of solid organ malignancies. BIA-ALCL has been reported in at least two patients with Li–Fraumeni syndrome, a hereditary cancer syndrome characterised by germline *TP53* mutations [21,22]. Somatic mutations of *TP53* have also been reported in the presence of *STAT3* mutations and are reported in around 12% of BIA-ALCL, despite being an uncommon finding in peripheral T-cell lymphoma [8].

Prior history of breast cancer is frequently seen in patients with BIA-ALCL, with subsequent mastectomy a common indication for breast implantation. Nevertheless, there has been some reports of an increased risk of BIA-ALCL in patients with germline mutations of *BRCA1* or *BRCA2* [23].

### 2.4. Structural Variants

Unlike systemic ALCL, structural variants are not commonly seen in BIA-ALCL. Indeed, there have been no cases of BIA-ALCL so far that have demonstrated *ALK*, *DUSP22* or *TP63* rearrangements. Of note, a STAT3-JAK2 fusion t(17;9)(q21.1) has been reported in one patient who also had *TP53* variant. The STAT3-JAK2 fusion has also been seen in T-cell lymphoproliferative disorders of the gastrointestinal tract [9].

### 2.5. Copy Number Variation and Cytogenetic Studies

Cytogenetic studies by conventional karyotyping are limited. Complex karyotypes have been reported in several series with no translocations characteristic of lymphoma found [24,25]. Los-de Vries et al. demonstrated characteristic loss of chromosome 20 in BIA-ALCL [11]. Shallow whole genome sequencing was performed on samples from twenty-nine patients and the most frequent copy number aberrancies (CNA) detected were gain of chromosome 2p (48%) and losses of 8p (48%), 20p (48%), and 20q (66%), which have been reported in other series [8,9]. The loss of 20q appears to be specific to BIA-ALCL and rarely reported in other ALCLs or PTCL-NOS. Moreover, the copy number load was higher in seroma BIA-ALCL in comparison to tumour, which is suggestive of greater intertumoural heterogeneity and that progression to invasive disease is by subclone selection [11]. Whole genome copy number analysis by Blombery et al. of thirteen patients revealed recurrent copy number loss of 1p21–22 (5/13) with a minimal deleted region containing the tumour suppressor gene *RPL5*. Deletions of *RPL5* are thought to result in ribosomal stress and are frequently seen in multiple myeloma. RPL5 is also negatively associated with MYC expression, with increased MYC expression demonstrated in RPL5 knockdown cell lines. Indeed, BIA-ALCL cells stain for MUM1 (IRF4) in most cases, suggesting MYC dysregulation, and focal amplification of MYC has been observed [7,26]. Recurrent losses of *PRDM1*, which encodes BLIMP-1, also known as PRDM1, a key regulator of plasma cell differentiation, were seen in three cases while focal amplification in *TNFRSF11A*, which encodes RANK, was seen in two patients [7]. RANK is ligated by RANKL, resulting in the activation of NF-κB, leading to downstream growth signalling [27]. RANKL is expressed by normal breast tissue where it activates mammary tissue, and alterations in the RANK pathway have been implicated in the carcinogenesis of breast cancer [28,29]. Laurent et al. reported CNA in eight patients, which demonstrated frequent complex chromosomal abnormalities. Recurrent gains in chromosomes 2, 9p, 12p, and 21 and losses on 4q, 8p, 15, 16, and 20 were reported with the JAK/STAT pathway and epigenetic regulator genes frequently affected by CNA. Deletion of 17p resulting in monoallelic loss of *TP53* was seen in three patients [8].

PDL1, encoded by *CD274*, is an immune checkpoint protein upregulated on cancer cells [30]. Tabanelli et al. utilised immunohistochemistry and fluorescent in situ hybridisation to demonstrate frequent PDL1 expression (56% of cases) and *CD274* CNA at 9p24.1 (33%), with all *CD274* CNA seen in cases with PDL1 expression [31]. This suggests an active PDL1/PD1 immune evasion pathway driven in part by alteration of 9p.

### 2.6. T-cell Receptor Rearrangements

Clonal T-cell receptor (TCR) rearrangements are frequently demonstrated in T-cell lymphomas and are consistently observed in BIA-ALCL [8,15]. Despite this demonstrated clonality, there is a lack of TCR surface expression when assessed by immunohistochemistry in a similar way to sALCL [32]. It is likely that other key growth and survival pathways are more critical than the T-cell receptor pathway in BIA-ALCL lymphogenesis.

### 2.7. Gene Expression Profiling

Gene expression profiling (GEP) studies have demonstrated molecular signatures specific for sALCL that are distinct from other PTCL [12]. Common and distinct pathways between ALK-positive ALCL and ALK-negative ALCL have also been characterised with GEP [33]. GEP studies have shown that BIA-ALCL shares a molecular profile with activated CD4+ T-cells. There is upregulation of chemotaxis genes *CCR6*, *MET*, and *CXCL14* when compared to normal CD4+ T-cells and viral transcription genes *RPS1*, *RPL17*, *RPS29* and *RPL18A* compared to other types of PTCL. *RPS10* is involved in ribosomal biogenesis and was one of the most differentially expressed genes between BIA-ALCL and other PTCL. Activation of STAT3 signalling pathways was seen with the downregulation of the T-cell receptor signalling pathway [34].

## 3. The Relationship between Pathogenesis and the Altered Genome

The sequence of events leading to the development of BIA-ALCL is yet to be defined but a proposed mechanism is a polymicrobial bacterial trigger, with bacterial biofilm attaching and forming on the surface of breast implants and leading to an inflammatory microenvironment [35]. This theory is supported by in vitro and in vivo studies linking implants with surface texture to higher levels of bacterial growth [36] and by the higher risk of developing BIA-ALCL associated with highly textured devices [37]. RNA sequencing demonstrated a significant upregulation of hypoxia signalling genes with carbonic anhydrase 9 (*CA9*) in particular, a biomarker of hypoxia-related malignancy, demonstrated to be overexpressed in hypoxic BIA-ALCL cell lines. This finding was unique to BIA-ALCL, with *CA9* overexpression relatively absent in other ALCLs, and suggests that a hypoxic microenvironment contributes to oncogenesis [38].

## 4. Implications for Targeted Therapy

Despite most patients presenting with localised disease able to be managed without systemic therapy, patients with advanced disease are typically treated with systemic chemotherapy following the same treatment principles of systemic ALK-negative sALCL [39]. Patients that relapse and require salvage therapy are similarly likely to be treated with the same principles and benefit from agents such as brentuximab-vedotin [40]. The frequent detection of aberrant JAK/STAT signalling in BIA-ALCL does provide an attractive potential therapeutic target. Pre-clinical data have demonstrated the anti-tumour effect of the JAK2 inhibitor ruxolitinib in a xenograft model of BIA-ALCL and the JAK/STAT-targeted tyrosine kinase inhibitor sunitinib in vitro [24,41]. Furthermore, the demonstration of an active PDL1/PD1 axis may represent a pathway to a novel treatment approach with PD1 or PDL1 inhibitors, while the prevalence of epigenetic dysregulation provides preclinical rationale for the use of ‘epigenome targeting’ agents such as HDAC inhibitors and DNA demethylating agents.

## 5. Discussion

**Figure 1:**
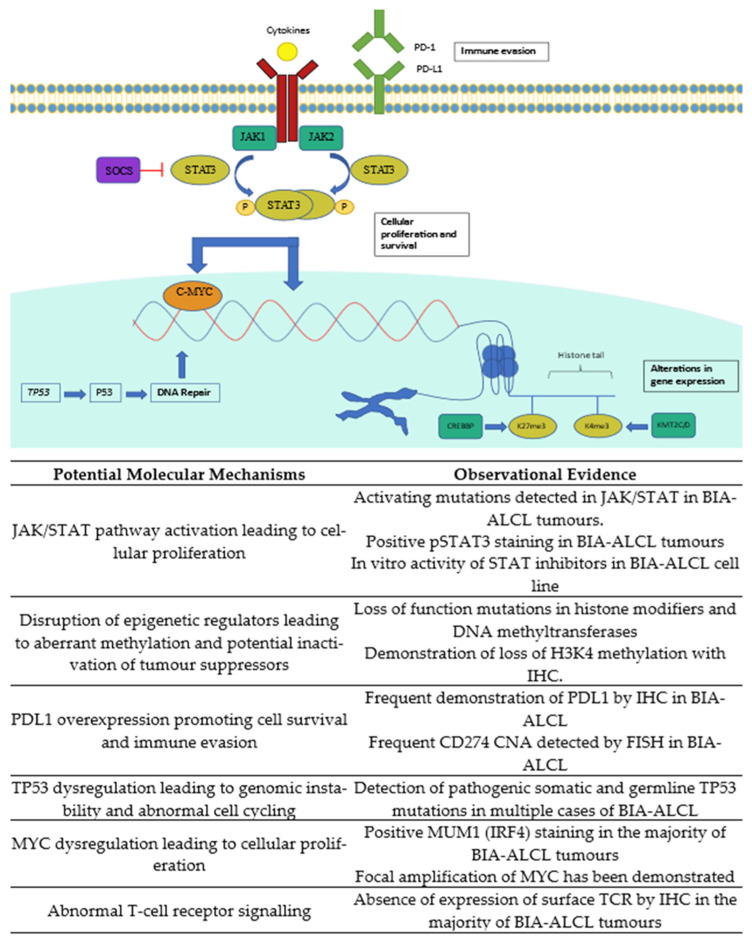
Summary of the molecular pathways potentially contributing to lymphogenesis. IHC, immunohistochemistry; CNA, copy number aberrations; FISH, fluorescent in situ hybridisation.

Genomic characterisation of BIA-ALCL demonstrates multiple potential pathogenic pathways (Figure 1). Comparison with other ALCLs is difficult due to limited large studies with complete sequencing data, but findings seemingly unique to BIA-ALCL have been demonstrated. The recognition of molecular variants in BIA-ALCL may not only aid in diagnosis and potentially novel treatment of refractory or relapsed systemic disease but may also provide another model for ‘environmentally-triggered’ genetic change contributing to lymphogenesis.

Although JAK/STAT pathway mutations are seen in other types of ALCL, the high frequency of alterations in BIA-ALCL may be explained by a shared pathway to lymphogenesis through common external factors. Frequent demonstration of STAT3 phosphorylation, even in the absence of detectable JAK/STAT pathway mutations, suggests constitutive STAT activation that may be driven directly or indirectly by chronic inflammation. Indeed, chronic inflammation has been demonstrated to lead to JAK/STAT pathway mutations in other cancers, and upregulation of interleukin-6-mediated STAT3 activation has been reported in BIA-ALCL cell lines [24,42].

The enrichment of mutations in epigenetic modifiers is not unique to BIA-ALCL, with epigenetic dysregulation common across various subtypes of PTCL where they contribute to oncogenesis in varying degrees. The presence of aberrancies in particular chromatin modifiers may help further distinguish BIA-ALCL from the other ALCLs, with further work needed to determine if particular epigenetic changes modify clinical behaviour [43]. Germline mutations in tumour suppressor genes have been reported with further data needed to delineate their contribution. The frequency of germline *TP53* mutations in individuals with breast implants is not defined so the implications are unclear, but this topic warrants ongoing consideration given the propensity for malignancy.

## 6. Conclusions

The genomic characterization of BIA-ALCL has demonstrated that despite similarities to the other ALCLs it is a unique entity. The ongoing characterization of molecular aberrancies may have diagnostic and prognostic implications but perhaps may also provide insight into the effect the environment has on the genome in the pathway to malignancy.

## Figures and Tables

**Table 1:** Summary of key sequence variants reported in BIA-ALCL case-series.

Study	No. of Cases	Methodology	Sequence Variants in JAK/STAT Pathway (% of Patients; Genes Containing Variants)	Sequence Variants in Epigenetic Regulators (% of Patients; Genes Containing Variants)	Other Genes of Interest
Di Napoli et al. [10]	5	Targeted sequencing (465 gene panel)	20% (*SOCS1*, *STAT3*)	20% (*DNMT3A*)	*TP53* (1 case)
Blombery et al. [7]	11	Targeted sequencing (180 gene panel) WES (2 cases)	91% (*JAK2*, *STAT3*)	9% (*SETD2*)	*TP53* (2 cases), *PTPN1* (1)
Oishi et al. [5]	15	Targeted sequencing of *JAK1*, *JAK3*, *STAT3*, *STAT5A*, *STAT5B*	27% (*JAK1*, *STAT3*)	Not assessed	Not assessed
Quesada et al. [9]	9	Targeted sequencing (400 and 199 genes)	78% (*JAK1*, *STAT3*, *SOCS1*, *STAT5B*)	78% (*SMARCB1*, *KDM5C*, *TET2*, *TET3*, *ARID4B*, *KDM6A*, *KMT2C*, *KMT2B*)	*TP53* (1 case), *PIK3CA* (1), *AXIN1* (1), *GNAS* (1)
Laurent et al. [8]	34	WES (22 cases) Targeted sequencing (400 gene panel) (24 cases)	59% (*STAT3*, *JAK1*, *SOCS3*, *STAT5B*, *PTPN1*, *SOCS1*)	74% (*KMT2C*, *CHD2*, *CREBBP*, *KMT2D*, *CHD8*, *DNMT3A*, *KDM1A*, *NCOR1*, *SUZ12*, *ARID2*, *ASXL3*, *HDAC2*, *HDAC4*, *HDAC5*, *HDAC8*, *TET2*)	*TP53* (4 cases), *EOMES* (4), *PTPN11* (2), *PIK3CG* (1), *CDKN2A* (1)
Los-de Vries et al. [11]	29	sWGS (29 cases) WES (7 cases)	43% (3/7 cases) (*STAT3*, *JAK1*)	29% (2/7cases) (*KMT2C*)	*MEF2A* (1 case)

WES, whole exome sequencing; sWGS, shallow whole genome sequencing.
